# Perceived social support and social/non-social problematic smartphone use among Chinese university students: a cross-sectional study of indirect associations via social anxiety

**DOI:** 10.3389/fpsyg.2026.1767558

**Published:** 2026-02-26

**Authors:** Xin Liu, Soongyu Kim, Ayeong Jang, Minghong Li, Jiayu Li

**Affiliations:** 1 Department of Social Welfare, Jeonbuk National University, Jeonju, Jeonbuk-do, Republic of Korea; 2 Office of Academic Affairs, Meizhouwan Vocational Technology College, Putian, Fujian, China; 3 Department of Media and Communication Studies, Jeonbuk National University, Jeonju, Jeonbuk-do, Republic of Korea

**Keywords:** Chinese university students, mediation, non-social PSU, perceived social support, problematic smartphone use, social anxiety, social PSU, structural equation modeling

## Abstract

**Introduction:**

Problematic smartphone use (PSU) is associated with poorer sleep, impaired attention, reduced academic functioning, and mental and physical health risks. This concern is salient among Chinese university students with smartphone engagement and elevated social anxiety (SA). Prior research links perceived social support (PSS) to lower anxiety and links SA to PSU severity, yet direct PSS–PSU associations are often modest or mixed. Moreover, most studies treat PSU as a unitary construct or examine subtypes, leaving subtype-specific tests of the PSS → SA → PSU pathway limited. Therefore, this study examined whether PSS was indirectly associated with social PSU (SPSU) and non-social PSU (NSPSU) via SA among Chinese university students.

**Methods:**

We conducted a cross-sectional self-report survey among undergraduates from one university in Jiangsu, China (*N* = 248; 58.1% women, 41.9% men; *M*age = 19.69 years, SD = 1.53). Structural equation modeling (SEM) with maximum-likelihood estimation and bootstrapping (5,000 resamples) was used to estimate indirect associations adjusting for gender, age, and subjective socioeconomic status. Common method variance was assessed using Harman’s single-factor test.

**Results:**

The structural model showed marginally acceptable fit (CFI = 0.90; TLI = 0.89; RMSEA = 0.07 [90% CI = (0.06, 0.08)]; SRMR = 0.07). PSS was negatively associated with SA (*β* = −0.26, *p* = 0.002). SA was positively associated with SPSU (*β* = 0.51, *p* = 0.001) and NSPSU (*β* = 0.55, *p* < 0.001). Bootstrapped estimates indicated significant indirect associations from PSS to SPSU and NSPSU via SA [SPSU: *β*_ind = −0.13, BC 95% CI (−0.36, −0.07); NSPSU: *β*_ind = −0.14, BC 95% CI (−0.39, −0.07)]. Direct paths from PSS to SPSU and NSPSU were non-significant; the PSS → SPSU direct effect was opposite in sign to the indirect effect. Harman’s test suggested limited common method bias (first factor = 29.124%).

**Conclusion:**

Social anxiety was associated with both PSU subtypes and was more strongly linked to NSPSU than SPSU in this sample. Perceived social support was indirectly associated with lower PSU through lower social anxiety, whereas direct associations with PSU subtypes were not significant. Given the cross-sectional design, findings reflect theory-consistent associations rather than causal effects.

## 1 Introduction

With the widespread adoption and enhanced functionality of smartphones, smartphones have become indispensable tools in daily life. China currently has approximately 1.1 billion mobile internet users, with 99.7% of users engaging in online activities via mobile devices. University students constitute a significant proportion of this demographic (China Internet Network Information Center, 2024). Over 80% of university students exhibit mobile phone dependency (China News Service, 2018), with 56% using their phones for more than 8 h daily (Chinese Sleep Research Society, 2024). Excessive mobile phone use may lead to problematic smartphone use (PSU), negatively impacting attention, sleep (Demirci et al., 2015; Wang et al., 2024a,b), academic performance (Sunday et al., 2021), and physical and mental health (Sohn et al., 2019). Therefore, it is necessary to understand the complex processes underlying PSU among university students, as such insights can provide important theoretical and practical implications for preventing problematic smartphone use in this population.

### 1.1 Perceived social support and social anxiety

Social support is typically regarded as an informational resource that enables individuals to believe they are cared for, respected, and belong to a social network of reciprocal obligations (Cobb, 1976). Building upon this, the perceived social support (PSS) examined in this study refers to an individual’s subjective sense of support and satisfaction derived from accessible social resources such as family, friends, and significant others (Zimet et al., 1988). Perceived social support is a stronger predictor of psychological states than objectively measured social support (Barrera, 1981).

Social anxiety (SA) denotes the anxiety experienced by individuals in real or imagined social situations, arising from anticipated or actual personal evaluations (Schlenker and Leary, 1982). Key characteristics include fear of negative evaluation/scrutiny (Rapee and Heimberg, 1997), safety behaviors and performative compensation (Amir and Bomyea, 2010), and significant physiological arousal and subjective discomfort (Stein, 2015).

Cohen and Wills (1985) distinguished two theoretical models explaining the relationship between social support and health: the buffering hypothesis and the main-effect model. In particular, the buffering hypothesis posits that social support primarily mitigates the adverse effects of stress on physical and mental health by moderating the relationship between stress and health outcomes (e.g., anxiety, depression, psychological distress). Based on this model, numerous studies have examined PSS as a moderator variable in the relationship between stress (or negative events) and negative mental health outcomes like depression or anxiety, validating its buffering effect (Martins et al., 2025; Padmanabhanunni et al., 2023; Szkody et al., 2021).

In contrast, the main-effect model suggests that even in the absence of overt stressors, higher levels of social support are linked to greater subjective well-being, self-esteem, and a sense of security, while lowering the risk of anxiety and other negative outcomes (Cohen and Wills, 1985). From the perspective of the main-effect model, PSS is often defined as a basic protective resource influencing individual mental health. Studies across different populations (e.g., general adults, university students, adolescents) have found that higher levels of PSS are associated with lower levels of anxiety symptoms and often show a significant direct negative effect in multivariate models (Lin et al., 2021; Pan et al., 2022). This provides a theoretical foundation for the assumption of the present study that perceived social support directly influences negative mental health outcomes such as anxiety.

The university stage represents a distinct developmental period marked by academic, social, and identity pressures. Compared to non-student peers, university students may face heightened anxiety risks. Existing research indicates that PSS serves as an important protective factor for university students’ mental health. When PSS is limited, individuals may be more likely to experience feelings of threat and self-doubt in social contexts, which can be expressed as anxiety and avoidance behaviors (Li et al., 2025; Scardera et al., 2020). Empirical studies among college samples have found that PSS from family, friends, significant others, or intimate partners is significantly negatively correlated with SA—that is, higher PSS correlates with lower SA (Chen et al., 2025). Studies involving Chinese university students also indicate that teachers’ emotional support, along with formal support from counseling centers and guidance institutions, plays a crucial role in reducing student anxiety and improving mental health (Fan and Liu, 2024; Li and Peng, 2021; Li Y. et al., 2023). Consistent with this resource-based view, perceived social support has been linked to stronger psychological resources. For example, in Turkish undergraduates, perceived social support was indirectly associated with lower COVID-19 uncertainty via resilience and academic self-efficacy (Green et al., 2024). Although SA was not measured, the findings illustrate a resource-building pathway through which perceived support may help individuals cope with pandemic-related uncertainty.

### 1.2 Social anxiety and different types of problematic smartphone use

PSU is commonly defined as maladaptive smartphone use accompanied by functional impairment and features resembling those observed in substance use disorders (e.g., tobacco, alcohol, and drugs). Such features include tolerance, withdrawal-like symptoms when use is stopped, continued use despite awareness of adverse consequences, and difficulties in controlling use (Billieux et al., 2015). A range of studies and reviews also indicates a robust positive association between anxiety and overall PSU severity (Elhai et al., 2017; Elhai et al., 2019; Xiao and Huang, 2022).

The Compensatory Internet Use Model (CIUM; also known as CIUT) posits that internet use serves as a coping strategy to escape real-world problems or alleviate negative emotions (e.g., stress, social anxiety). Related work has also examined resource-buffering patterns in fear-related pathways to smartphone addiction; for example, fear of COVID-19 was positively associated with smartphone addiction among Turkish adolescents, partly via lower resilience (Yıldırım and Çiçek, 2022). While individuals may experience short-term emotional relief, this can lead to negative consequences of excessive dependence (Kardefelt-Winther, 2014). Existing studies based on the CIUM have confirmed that SA is one of the key predictors of PSU (Elhai et al., 2018; Wolniewicz et al., 2018).

In recent years, the I-PACE (Interaction of Person-Affect-Cognition-Execution) model (Brand et al., 2016) has provided a core theoretical framework for explaining mobile phone dependency behavior (Elhai et al., 2020a,b; Wegmann et al., 2017). The model treats the internet primarily as a medium rather than an addictive substance and emphasizes application-specific pathways. Accordingly, different online activities are expected to vary in usage motivations, reinforcement patterns, and symptom presentations. This perspective supports refining the broad notion of “generalized internet addiction” into “specific internet use disorders” (Brand et al., 2016). Research on PSU should primarily focus on specific application types (e.g., social networking, short-video, gaming, and information-seeking apps) rather than broadly examining the overall phenomenon (Marino et al., 2021). In line with this perspective, the Uses and Gratifications Theory (UGT) emphasizes the proactive nature of media use, positing that individuals select different types of media content based on their psychological and social needs (Katz et al., 1973). Prior work therefore often distinguishes social uses related to online social interaction and relationship maintenance (e.g., voice/video calls, instant messaging, social networking) from non-social uses associated with relaxation, information-seeking, and entertainment (e.g., web browsing, gaming, video and music viewing) (Elhai et al., 2016; Elhai et al., 2020b; Van Deursen et al., 2015). Because smartphone functions are highly integrated, these two categories may intersect and overlap in real-world contexts (Elhai et al., 2016).

Existing research has examined the relationship between SA and both social problematic smartphone use (SPSU) and non-social problematic smartphone use (NSPSU). On the one hand, systematic reviews and meta-analyses indicate that individuals with higher levels of social anxiety are more likely to develop SPSU, with a stable positive correlation existing between SA and SPSU (Wu et al., 2024). Individuals with social anxiety, constrained by perceived offline social threats and efficacy limitations, often prefer online social interactions to gain a sense of control and security (Davis, 2001; O’Day and Heimberg, 2021). Conversely, SA is also closely linked to NSPSU. Anxiety drives individuals to engage more frequently in non-social activities like gaming, scrolling through short videos, or passively browsing information for “emotional regulation” (escaping or alleviating negative emotions). This pattern is particularly evident in conditions such as Internet Gaming Disorder, Problematic Internet Use, and problematic binge-watching behavior (Ding et al., 2023). From a UGT perspective, short-video and feed-based apps enable rapid distraction and low-effort, user-controlled consumption (e.g., endless scrolling of short clips or news feeds). For individuals higher in social anxiety, these functions may support avoidance-based emotion regulation without the demands of real-time interaction or fear of evaluation. Consistent with this rationale, prior studies typically report positive links between social anxiety and both SPSU and NSPSU, with NSPSU sometimes showing stronger associations—especially for passive entertainment and information-consumption use. However, most work has examined SPSU and NSPSU separately, so direct comparisons remain limited and subtype-specific intervention guidance is still underdeveloped.

### 1.3 Mediating role of social anxiety

Existing research indicates that negative emotional experiences may constitute a crucial psychological mediating mechanism in the relationship between PSS and PSU. For instance, depression and loneliness have been demonstrated to mediate the association between social support and PSU (Peng et al., 2022; Yang et al., 2023). Mechanism-oriented evidence in university samples also supports the plausibility of indirect associations. For example, in Turkish university students, perceived social support was suggested to mediate the links between problematic social media use and life satisfaction as well as depressive symptoms (Çiçek et al., 2024). However, this cross-sectional evidence does not establish causality and does not test social anxiety–related mechanisms. Accordingly, whether social anxiety operates as a key mediator linking PSS to PSU remains unclear. Furthermore, studies indicate that SA also partially mediates the relationship between perceived stress and PSU (Liu and Han, 2025). When individuals experience insufficient fulfillment of basic psychological needs or weaker positive psychological resources, this may also increase PSU tendencies through multiple mediating pathways such as social anxiety and loneliness (Li D. et al., 2023; Sun et al., 2023).

Based on the social support main-effect model, higher PSS may alleviate feelings of social threat and self-doubt and thus reduce SA (Cohen and Wills, 1985). Empirical studies also report a significant negative association between PSS and SA (Chen et al., 2025). From the CIUM perspective, individuals with high SA may be more likely to engage in compensatory smartphone use for emotional relief or vicarious interaction, which can elevate the risk of PSU (Elhai et al., 2018; Kardefelt-Winther, 2014; Wolniewicz et al., 2018). Prior evidence likewise supports a robust positive association between SA and PSU (Elhai et al., 2017; Elhai et al., 2019; Xiao and Huang, 2022).

Furthermore, research has found that the direct correlation between PSS and PSU is often weak or even unstable, while indirect pathways mediated by variables such as negative emotions may be more pronounced. For instance, studies involving Turkish and Chinese university students indicate a weak yet significant negative correlation between PSS and overall PSU levels, with PSS’s influence on PSU likely mediated by psychological factors (Ding et al., 2022).

Based on this, a plausible pathway can be proposed: PSS may indirectly influence both types of PSU through its effect on SA, with SA mediating the relationship between PSS and SPSU on one hand, and between PSS and NSPSU on the other. A study by Çelik and Konan (2019) using Turkish pre-service teachers as participants directly supports this hypothesis, revealing that interactional anxiety fully mediates the “PSS → PSU” relationship. However, direct empirical tests of this mediating mechanism remain extremely limited, and there is a lack of empirical evidence specific to Chinese university students that distinguishes between different types of PSU. To clarify the proposed mechanism, we conceptualize PSS as a psychosocial resource (main-effect model) that may relate to lower SA; in turn, elevated SA may increase reliance on smartphone-based activities for affect regulation and avoidance (CIUM/I-PACE), thereby elevating PSU risk. Given the heterogeneity of smartphone activities, we distinguish social versus non-social PSU based on UGT and application-specific accounts, expecting social anxiety to be relevant to both but potentially more strongly to non-social, low-interaction coping uses (e.g., passive scrolling, short-video viewing, gaming).

### 1.4 Research hypotheses and model

Research on university students suggests that higher PSS, including informal support from family, friends, and significant others and, when assessed, formal support from school/community institutions, is associated with lower SA. PSS has also been linked to lower PSU, although direct PSS–PSU associations are often modest or mixed, and indirect pathways via negative affect have been more consistently supported. In contrast, SA has been consistently associated with higher PSU.

Despite this evidence, several limitations remain. First, prior PSU research has often conceptualized PSS as a stress-buffering moderator. PSS remains underexamined as a direct psychosocial resource and in the mechanisms through which it may relate to PSU. Second, many studies rely on PSU total scores or other aggregate indicators, which may obscure pathways leading to distinct PSU subtypes. Third, direct tests of the PSS → SA → PSU subtype mediation pathway among Chinese university students remain limited.

Because smartphones are multifunctional, PSU is unlikely to be a homogeneous construct. To capture subtype-specific mechanisms, we distinguish two forms of PSU: social (SPSU) and non-social (NSPSU). At present, it remains unclear whether the same psychosocial pathway (PSS → SA) operates similarly across these PSU subtypes, or whether the strength of associations differs when the subtypes are modeled simultaneously. Using a sample of Chinese university students, we integrate PSS, SA, and the two PSU subtypes into a unified analytical framework: we model PSS as an upstream psychosocial resource (main-effect model) with a direct association with SA, and test whether this pathway operates similarly for SPSU versus NSPSU. CIUM specifies the motivational premise for compensatory use (negative affect → coping-motivated use), whereas I-PACE delineates how affective states may translate into application-specific problematic use through person-related vulnerabilities and cognitive–executive processes. Guided by UGT and application-specific accounts, we examine the direct associations of PSS with SPSU and NSPSU and the indirect associations via SA. Therefore, we propose the following hypotheses:

*H1*: PSS and SA are negatively correlated.

*H2a*: SA and SPSU are positively correlated.

*H2b*. SA and NSPSU are positively correlated.

*H3a*: SA mediates the association between PSS and SPSU.

*H3b*: SA mediates the association between PSS and NSPSU.

Previous research indicates that females, individuals with lower subjective socioeconomic status (SSES), and younger individuals typically exhibit higher anxiety symptoms and more severe PSU (Dashiff et al., 2009; McLean et al., 2011; Van Deursen et al., 2015). Thus, gender (1 = male, 2 = female), age, and SSES were included as control variables.

## 2 Methods

### 2.1 Study population

This study employed convenience sampling to recruit Chinese university students who participated voluntarily. An electronic questionnaire was distributed to students at one university in Jiangsu Province, China, via the online survey platform Wenjuanxing between November 6 and 14, 2025. Before data collection, the study was approved by the Institutional Review Board of Jeonbuk National University (File No. JBNU IRB 2025–09–001-004), and all participants provided electronic informed consent before completing the survey. To support online data quality, the survey was configured with mandatory responses to prevent missing data, and standard platform settings were used to reduce duplicate submissions (e.g., restricting multiple entries from the same device/IP where feasible).

Sample size considerations. We set an *a priori* analyzable target of *N* ≥ 200 for SEM, acknowledging that required *N* varies with model characteristics and evaluation criteria and should be viewed as a pragmatic minimum for a modest-complexity model rather than a universal rule (Westland, 2010; Wolf et al., 2013). Because mediation power depends on the magnitudes of the constituent paths and the test used—and may require several hundred cases when a component path is small—we additionally conducted a supplementary regression-type power check (Fritz and MacKinnon, 2007). G*Power 3.1 indicated that detecting a small effect (Cohen’s f^2^ = 0.05) with *α* = 0.05 and 80% power with four predictors requires *N* ≈ 244; thus, the final analyzable sample (*N* = 248) meets this benchmark, and we oversampled (≥ 300 responses) to allow for pre-specified data-quality exclusions (Faul et al., 2009).

A total of 334 questionnaires were submitted. We applied pre-specified data-quality screens and excluded responses that met any of the following criteria: (1) straight-lining across all items in at least two core scales; (2) completion time below the 5th percentile of the sample distribution (< 102 s); or (3) self-reported age < 18 years. These exclusions removed 86 cases (25.7%), leaving 248 valid responses for analysis (74.3%). Although the sample was volunteer-based and drawn from a single university, the study was designed to be exploratory and hypothesis-generating; broad campus recruitment and pre-specified data-quality screens were used to reduce invalid responding.

The mean age of participants was 19.69 years (SD = 1.53). By gender, 104 participants (41.9%) were male and 144 (58.1%) were female. Regarding SSES, 7 students (2.8%) rated themselves as “very poor,” 46 (18.5%) as “poor,” 171 (69.0%) as “average,” 19 (7.7%) as “good,” and 5 (2.0%) as “very good,” with “average” being the predominant overall level. Grade distribution showed 100 freshmen (40.3%), 44 sophomores (17.7%), 58 juniors (23.4%), and 46 seniors or above (18.5%). The sample covered all grades but was slightly skewed toward lower-year students. Detailed demographic characteristics are presented in Table 1.

**Table 1:** Demographic characteristics of study participants (*N* = 248).

Variable	Category	*n*	%
Gender	Male	104	41.9
	Female	144	58.1
Subjective socioeconomic status (SSES)	Very poor	7	2.8
	Poor	46	18.5
	Average	171	69.0
	Good	19	7.7
	Very good	5	2.0
Grade	Freshman	100	40.3
	Sophomore	44	17.7
	Junior	58	23.4
	Senior and above	46	18.5

### 2.2 Tools

#### 2.2.1 Modified multidimensional perceived social support scale (MSPSS)

Building upon the Multidimensional Perceived Social Support Scale (MSPSS; family, friends, and significant others) (Zimet et al., 1988), this study added dimensions of “school support” and “community institutional support” tailored to the context of Chinese university students, resulting in a 23-item scale. To maintain consistency with the full questionnaire and reduce respondent burden, the original 7-point Likert scale was modified to a 5-point scale (1 = Strongly Disagree, 5 = Strongly Agree). Higher scores indicate stronger perceived social support. Across the pretest and formal samples, item analysis and exploratory factor analysis (EFA)/ confirmatory factor analysis (CFA) suggested that the “school support” and “community institutional support” items aligned with the same factor. Accordingly, “school support” and “community institutional support” were merged into a single Formal Support factor in the main analyses. Items with suboptimal psychometric performance were then removed to improve the scale’s measurement quality. The final scale contains 19 items and comprises four subdimensions: Family, Friend, Significant Other Support, and Formal Support. The internal consistency of each subscale was satisfactory, with Cronbach’s *α* values of 0.88, 0.91, 0.93, and 0.96, respectively.

#### 2.2.2 Social interaction anxiety scale (SIAS) and social phobia scale (SPS) short forms (SIAS-6 and SPS-6)

Social anxiety was assessed using the brief forms of the Social Interaction Anxiety Scale and the Social Phobia Scale (SIAS-6 and SPS-6) (Peters et al., 2012). The Chinese versions were translated and validated by Ouyang et al. (2020). When used together, these scales have demonstrated good internal consistency and construct validity in a Chinese university student sample. The original short-form scales comprised 12 items. The SIAS-6 assessed anxiety in general social interaction settings (e.g., “I tense up if I meet an acquaintance on the street”), while the SPS-6 evaluated anxiety when performing tasks under others’ gaze (e.g., “When in an elevator, I am tense if people look at me”). In this study’s predictive analysis and CFA, two items exhibiting relatively weaker statistical performance and content representativeness were removed based on factor loadings, item-total correlations, and model residuals. The final scale comprises 10 items: 4 for the interaction anxiety factor (SIAS) and 6 for the performance anxiety under observation factor (SPS). All items were rated on a 5-point scale ranging from 0 (not at all true of me) to 4 (extremely true of me), with higher scores reflecting greater social anxiety in each subdomain. In this study, Cronbach’s *α* for the interaction anxiety and performance anxiety under observation subscales were 0.85 and 0.92, respectively.

#### 2.2.3 Mobile phone addiction type scale (MPATS)

SPSU and NSPSU were measured using the MPATS scale (Liu et al., 2022). The original scale comprised 26 items across four dimensions: social dependency, gaming dependency, information-seeking dependency, and short-video dependency. It assesses mobile phone addiction tendencies oriented toward different functional aspects. Based on factor loadings, item-total correlations, and model fit indices, three items with relatively weak statistical performance and high content overlap with other items were removed. The final version retained 23 items: social dependency (5 items), gaming dependency (5 items), information-seeking dependency (7 items), and short-video dependency (6 items). Items for each dimension were rated on a 5-point Likert scale (1 = never, 5 = always), and dimension scores were computed by averaging the corresponding items. Higher scores indicate more severe dependence in the corresponding dimension. Based on the UGT, this study combined the last three usage categories into the NSPSU model, which together with the SPSU formed two types of dependent variables. Cronbach’s α for the four dimensions in this study were 0.88, 0.91, 0.96, and 0.91, respectively.

### 2.3 Data analysis

Preliminary data analysis was conducted using SPSS 29.0. The questionnaire platform was configured with mandatory response requirements, resulting in no missing values across items. Mean scores for each variable dimension were calculated based on the final items confirmed through EFA and CFA for subsequent analysis. The variables in the research model exhibited near-normal distributions, with the maximum absolute skewness of the four core variables being 0.59 and the maximum absolute kurtosis being 0.21. Variance Inflation Factors (VIF) were calculated via linear regression to examine multicollinearity. The VIF values for all predictor variables ranged between 1.05 and 1.18, indicating no severe multicollinearity issues in the research model.

CFA and SEM were conducted using Amos 29.0. Before testing structural paths, measurement models for PSS, SA, and PSU were first established using item-level data, followed by item-by-item CFA. Since all items employed a 5-point Likert scale and the sample size exceeded 200, existing research generally treats such items as quasi-continuous variables and employs maximum likelihood (ML) estimation for parameter estimation. This study also employed the ML method, estimating parameters based on the covariance matrix from *N* = 248. To achieve model identification, one loading per factor was fixed at 1, while the remaining factor loadings were freely estimated. Residual covariances were fixed at 0 when lacking sufficient theoretical justification, and first-order factors were allowed to correlate. Model fit was assessed using the Comparative Fit Index (CFI) and the Standardized Root Mean Square Residual (SRMR). Models with CFI ≥ 0.95 and SRMR ≤ 0.08 are considered to fit well; models with CFI ≥ 0.90 and SRMR ≤ 0.10 are considered marginally acceptable (Hu and Bentler, 1999; Schermelleh-Engel et al., 2003).

**Figure 1:**
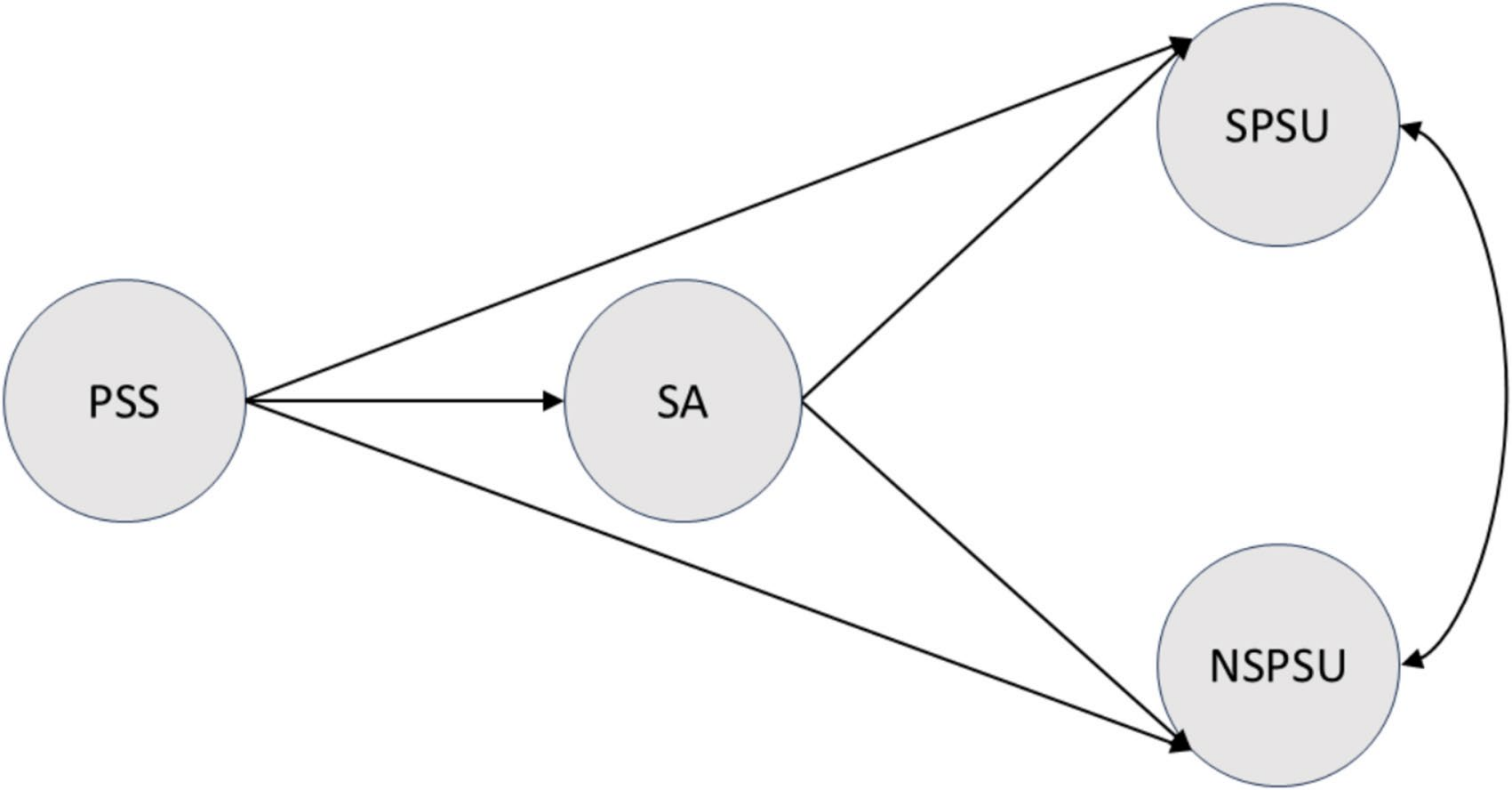
Hypothesized model. This path diagram illustrates the hypothesized relationships in which perceived social support is associated with social anxiety as well as social and non-social problematic smartphone use, and social anxiety, in turn, is associated with both social and non-social problematic smartphone use, with gender, age, and subjective socioeconomic status as covariates. Circles represent latent variables. PSS, perceived social support; SA, social anxiety; SPSU, social problematic smartphone use; NSPSU, non-social problematic smartphone use.

The structural model (see Figure 1) uses the PSS → SA path to test H1; SA → SPSU and SA → NSPSU to test H2a and H2b. Gender, age, and SSES were included as observed covariates, with paths to SA and both PSU outcomes. Standardized estimates and multiple correlations squared (*R*^2^) were used to assess effect sizes and variance explained.

Mediation effects were defined as the product of two direct path coefficients (*a* × *b*). To test the mediating role of SA between PSS and SPSU/NSPSU (Hypotheses H3a and H3b), this study employed the nonparametric bias-corrected bootstrap method in AMOS, conducting 5,000 bootstrap samples. The significance of the mediating effect was determined based on whether the 95% bootstrap confidence interval for the indirect effect included 0 (Hayes, 2017; MacKinnon et al., 2004). The bias-corrected bootstrap *p*-values were thus obtained.

## 3 Results

### 3.1 Common method bias

Given the self-reported, single-survey design, common method bias was assessed using Harman’s single-factor test. An unrotated exploratory factor analysis including all retained items indicated that nine factors had eigenvalues > 1, and the first factor accounted for 29.124% of the total variance (below the commonly used 40% criterion). This pattern suggests that common method variance is unlikely to be a major concern.

### 3.2 Descriptive results

**Figure 2:**
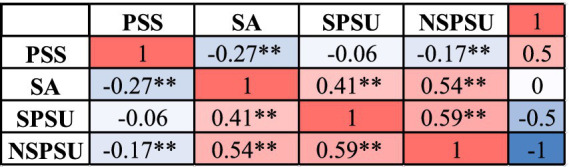
Correlation matrix of primary variables. Heatmap-style correlation matrix showing small-to-moderate negative associations between perceived social support and social anxiety, and moderate positive associations between social anxiety and both types of problematic smartphone use. PSS, perceived social support; SA, social anxiety; SPSU, social problematic smartphone use; NSPSU, non-social problematic smartphone use. **p < 0.01, *p < 0.05 (two-tailed).

Descriptive statistics for each variable are presented in Table 2, while correlations among key variables are shown in Figure 2. Overall, participants’ PSS scores were moderately high (*M* = 3.58, SD = 0.70), with a gradient across the four sources: “family support (*M* = 3.87) > friend support (*M* = 3.82) > support from significant others (*M* = 3.57) > formal support (*M* = 3.27).” SA scores were generally low overall (*M* = 1.27, SD = 0.87). SPSU scores were higher than NSPSU scores, with lower scores for gaming dependence (*M* = 1.98).

**Table 2:** Means and standard deviations for the primary variables.

Variables	*M*	SD
1. Perceived social support	3.58	0.70
1.1 Family support	3.87	0.84
1.2 Friend support	3.82	0.78
1.3 Support from significant others	3.57	0.96
1.4 Formal support (schools and community institutions)	3.27	0.93
2. Social anxiety	1.27	0.87
2.1 Interaction anxiety	1.22	0.85
2.2 Performance anxiety under observation	1.30	0.97
3. Social problematic smartphone use	3.01	0.91
4. Non-social problematic smartphone use	2.40	0.83
4.1 Gaming dependence	1.98	0.82
4.2 Information-seeking dependence	2.46	1.01
4.3 Short video dependence	2.68	0.95

Perceived social support is calculated as the average of scores across four sub-dimensions (formal support, friend support, family support, and significant other support). Non-social PSU is calculated as the average of scores across three sub-dimensions (gaming dependency, information-seeking dependency, and short-video dependency).

Correlation results showed that SA was moderately positively correlated with both types of PSU (with SPSU: *r* = 0.41, *p* < 0.001; with NSPSU: *r* = 0.54, *p* < 0.001). SPSU also showed a moderate positive correlation with NSPSU (*r* = 0.59, *p* < 0.001), indicating that students with higher social anxiety exhibit greater risk in both types of problematic smartphone use. PSS showed a small to moderate negative correlation with SA (*r* = −0.27, *p* < 0.001) and a weak negative correlation with NSPSU (*r* = −0.17, *p* < 0.01), while the correlation with SPSU was non-significant (*r* = −0.06, *p* = 0.36). This indicates that PSS is primarily associated with NSPSU rather than SPSU. Overall, SA was the variable most strongly linked to both types of PSU, while the direct associations between PSS and PSU were generally weak.

### 3.3 Confirmatory factor analysis (CFA) results

CFA was conducted on PSS, which comprises four subscales: family, friends, special others, and formal support. After item screening, 19 items were retained. All items loaded strongly on their intended subscales (standardized loadings = 0.73–0.93), supporting convergent validity. Inter-factor correlations were moderate (0.36–0.76), consistent with related yet distinguishable first-order dimensions that collectively reflect overall perceived social support. Model fit was marginally acceptable [*χ*^2^(146, *N* = 248) = 537.84, *p* < 0.001; CFI = 0.92; TLI = 0.90; RMSEA = 0.10, 90% CI = (0.10, 0.11); SRMR = 0.06]. Given the stable loadings and the theoretically hierarchical nature of social support, PSS was subsequently modeled in SEM as a single latent construct, using the mean scores of the four subscales as observed indicators. This approach aligns with common recommendations for parceling (Bagozzi and Heatherton, 1994), facilitating a more parsimonious and stably estimated SEM solution while preserving measurement validity.

During CFA of the two-factor structure (interaction anxiety and performance anxiety under observation) of the Social Anxiety Scale, standardized factor loadings for each item ranged from 0.72 to 0.84, indicating good convergent validity. The correlation coefficient between the two first-order factors was 0.84, suggesting high inter-factor correlation while maintaining distinguishability. The measurement model demonstrated good fit [*χ*^2^(34, *N* = 248) = 120.68, *p* < 0.001; CFI = 0.95; TLI = 0.93; RMSEA = 0.10, 90% CI = (0.08, 0.12); SRMR = 0.04]. Given this study’s focus on the mediating role of “overall SA” between PSS and different types of PSU, and the high correlation between the two subscales, SA was treated as a single latent variable in subsequent SEM analyses, with the mean score of the two subscales serving as its observed indicators.

The PSU was modeled as a multidimensional construct comprising SPSU and NSPSU. SPSU, as a first-order latent variable, is indicated by five items; NSPSU, as a second-order latent variable, is indicated by three first-order factors: gaming, information-seeking, and short video dependency. CFA results indicate that the standardized loadings of the second-order NSPSU on its three first-order factors range from 0.69 to 0.93, while the loadings of items under the first-order factors range from 0.54 to 0.91, all falling within the moderate to high range. The measurement model is marginally acceptable, approaching good fit [*χ*^2^(223, *N* = 248) = 511.45, *p* < 0.001; CFI = 0.94; TLI = 0.93; RMSEA = 0.07, 90% CI = (0.06, 0.08); SRMR = 0.06]. Composite reliability (CR) for both first-order and second-order NSPSU ranged from 0.83 to 0.96, with average variance extracted (AVE) between 0.51 and 0.75—both exceeding common thresholds, indicating strong convergent validity across dimensions. The correlation coefficient between SPSU and NSPSU was *r* = 0.69, satisfying the Fornell–Larcker criterion, indicating good discriminant validity between the two PSU types. Therefore, the multidimensional measurement structure of second-order NSPSU + first-order SPSU was retained in subsequent SEM analyses to distinguish different PSU types.

**Figure 3:**
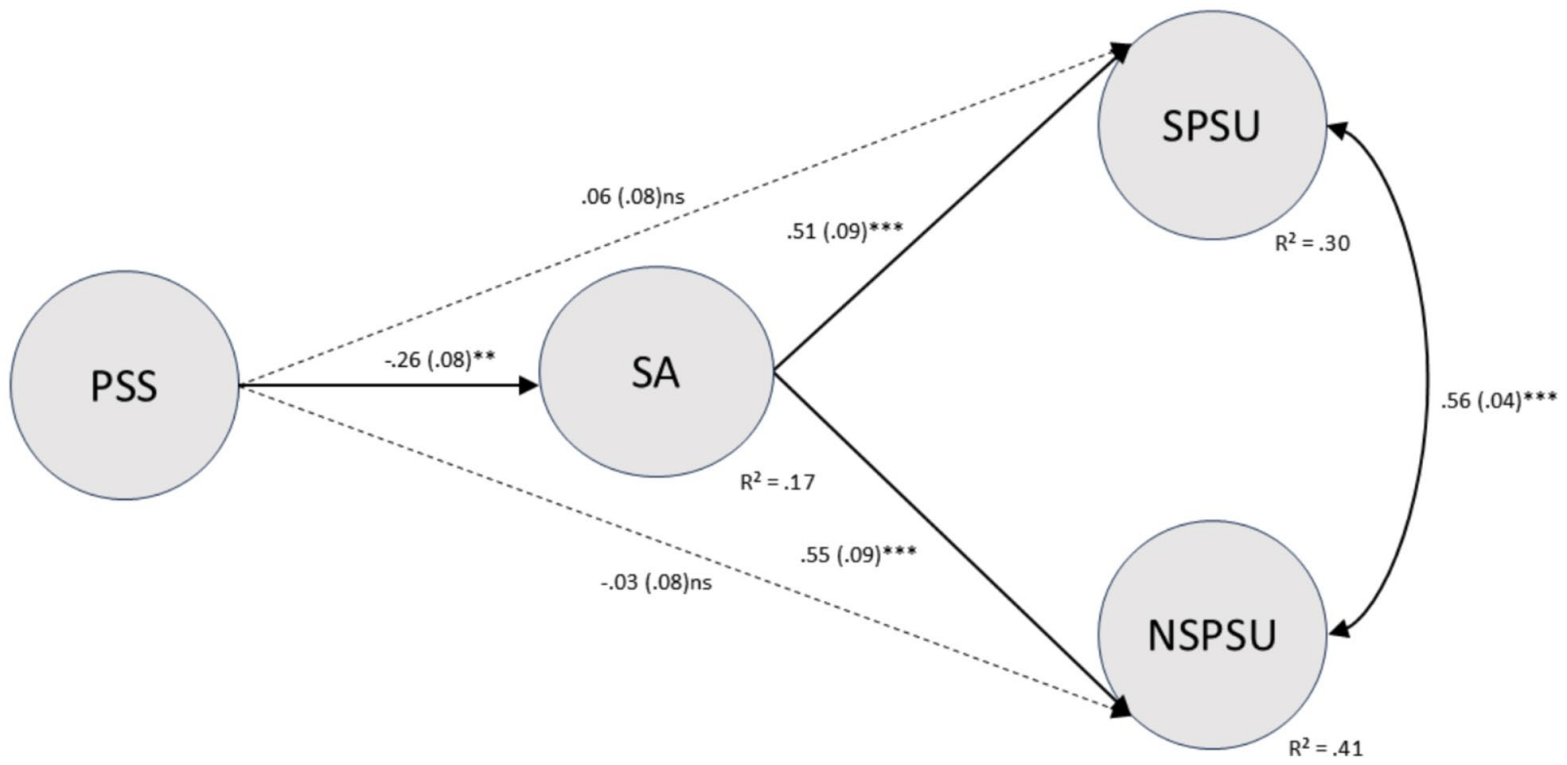
SEM model with standardized path coefficients. Final structural equation model with standardized path coefficients showing perceived social support negatively predicting social anxiety and social anxiety positively predicting both social and non-social problematic smartphone use; direct paths from perceived social support to both outcomes are small and not significant. Circles represent latent variables. PSS, Perceived Social Support; SA, Social Anxiety; SPSU, Social Problematic Smartphone Use; NSPSU, Non-social Problematic Smartphone Use. The numbers on the arrows indicate standardized regression coefficients *β*, with bootstrap standard errors (SE) in parentheses. After controlling for gender, age, and SSES, the model explains *R*^2^ variance in SA, SPSU, and NSPSU. * *p* < 0.05, ** *p* < 0.01, *** *p* < 0.001.

After controlling for gender, age, and SSES, the hypothesized model depicted in Figure 1 was tested. The overall model fit was marginally acceptable [*χ*^2^(441, *N =* 248) = 975.77, *p* < 0.001; CFI = 0.90; TLI = 0.89; RMSEA = 0.07, 90% CI = (0.06, 0.08); SRMR = 0.07].Figure 3 presents standardized path coefficients. Results indicated:

1. PSS significantly negatively predicted SA (*b* = −0.41, *SE* = 0.13, *p* = 0.002; *β* = −0.26), supporting H1: higher PSS was associated with lower SA.
2. SA significantly and positively predicted both types of PSU. The regression coefficient for SPSU was (*b* = 0.43, *SE* = 0.11, *p* = 0.001; *β* = 0.51), while the regression coefficient for NSPSU was (*b* = 0.51, *SE* = 0.10, *p* < 0.001; *β* = 0.55), supporting H2a and H2b and indicating a slightly stronger association between SA and NSPSU than with SPSU.
3. SSES exerted a small but significant negative predictive effect on SA (*b* = −0.19, *SE* = 0.08, *p* = 0.02; *β* = −0.17), indicating that better SSES was associated with lower SA. Its direct path to NSPSU was negative and near-significant (*b* = −0.11, *SE* = 0.06, *p* = 0.07; *β* = −0.11), suggesting students with poorer SSES may face a higher risk of NSPSU. Gender (2 = female) significantly and positively predicted SA (*b* = 0.34, *SE* = 0.10, *p* = 0.003; *β* = 0.24), and exerted a small but significant positive effect on SPSU (*b* = 0.19, *SE* = 0.09, *p* = 0.02; *β* = 0.16), indicating that female students exhibited slightly higher levels of social anxiety and SPSU tendencies than males after controlling for other variables. Age did not significantly influence SA or either type of PSU overall.

### 3.4 Mediational effects results

For NSPSU, the indirect effect of PSS via SA was significantly negative [*ab* = −0.21, SE = 0.08, *p* = 0.002, *β*_ind = −0.14, BC 95% CI (−0.39, −0.07)]. The direct effect of PSS → NSPSU was small and non-significant, while the total effect was significantly negative [*b*_total = −0.26, SE_total = 0.13, *p* = 0.03, BC 95% CI (−0.55, −0.02)]. This indicates that higher PSS primarily reduces NSPSU indirectly via SA. Together, these results are consistent with an indirect-only (predominantly indirect) pattern, suggesting that PSS was primarily linked to NSPSU through SA, supporting H3b.

For SPSU, PSS also exhibited a significant negative indirect effect via SA [*ab* = −0.18, *SE* = 0.07, *p* = 0.002, *β*_ind = −0.13, BC 95% CI (−0.36, −0.07)]. However, the direct effect of PSS → SPSU was marginally positive but not significant, resulting in a total effect of PSS on SPSU that was near zero and non-significant [*b*_total = −0.10, *SE*_total = 0.12, *p* = 0.35, BC 95% CI (−0.35, 0.13)]. Overall, the SPSU results indicate a significant indirect association via SA alongside a small, opposite-signed non-significant direct path (i.e., an inconsistent/suppression-like pattern), supporting H3a with respect to the indirect pathway.

## 4 Discussion

This study tested a theoretically specified model linking perceived social support (PSS), social anxiety (SA), and two forms of problematic smartphone use (PSU)—social PSU (SPSU) and non-social PSU (NSPSU)—in a cross-sectional sample of Chinese university students. By distinguishing social versus non-social PSU, we aimed to clarify whether the pattern of associations differs across PSU subtypes in this cultural context and to generate testable hypotheses for future longitudinal and intervention research.

Findings revealed a small to moderate significant negative correlation between PSS and SA, supporting Hypothesis H1. This aligns with the main-effect model of social support (Cohen and Wills, 1985), which posits that higher levels of perceived social support are typically associated with lower anxiety levels. The magnitude of the PSS–SA correlation (*r* = −0.27) in this study falls within the range of findings reported across culturally diverse college populations in prior research (*r* values ranging from −0.25 to −0.34) (Çelik and Konan, 2019; Moghtader and Shamloo, 2019).

SA showed moderate to strong positive correlations with both SPSU and NSPSU, and demonstrated substantive predictive power for both types of PSU in SEM, supporting H2a and H2b. Dependent correlation tests (Steiger, 1980) further revealed a significant difference between the two correlations (∣*t*∣ = 2.69, df = 245, *p* = 0.007), indicating that the relationship between SA and NSPSU is significantly stronger than that with SPSU. This finding aligns with existing research evidence showing “a significant positive correlation between anxiety and overall PSU levels” (Elhai et al., 2017; Elhai et al., 2019; Xiao and Huang, 2022) and aligns with findings on the relationship between SA and problematic social media use, online gaming, and problematic internet use (Ding et al., 2023; Wu et al., 2024). Overall, these findings suggest that SA may not only increase reliance on relatively “safer” online social interactions but also reinforce problematic non-social uses (e.g., gaming, short videos, news scrolling) through escapist and emotion-regulating usage patterns, thereby exhibiting stronger associations with NSPSU.

The stronger SA–NSPSU association warrants consideration. Among students high in social anxiety, non-social smartphone activities (e.g., short-video viewing, passive information scrolling, or gaming) may offer rapid, low-effort distraction and mood modification with minimal interpersonal exposure or evaluation threat. This low-interaction, avoidance-congruent regulation pattern may partly explain why SA was more strongly associated with NSPSU than SPSU in the present sample. Practically, these findings highlight social anxiety and maladaptive emotion regulation as plausible targets for reducing non-social, low-interaction coping uses of smartphones.

Because the survey was cross-sectional, the analyses speak to covariation rather than cause-and-effect. The reported “indirect effects” should therefore be read as theory-driven SEM estimates that decompose associations under the assumed ordering (PSS → SA → PSU), not as evidence that social anxiety functions as a causal mechanism. Establishing that direction will require designs that demonstrate temporal precedence (e.g., multi-wave longitudinal data) and/or experimental or intervention approaches.

In line with the hypothesized model, perceived support was inversely related to social anxiety, and social anxiety was positively related to both SPSU and NSPSU. Bootstrap results yielded statistically significant negative indirect associations from PSS to both PSU outcomes via SA: students who felt less supported tended to report greater social anxiety, and higher anxiety was in turn linked to more problematic smartphone use. For NSPSU, most of the overall association was carried through the indirect pathway, with the direct PSS → NSPSU link remaining small and non-significant. For SPSU, the direct path was weak and opposite in sign, partly offsetting the negative indirect pathway—an inconsistent (suppression-like) pattern. The inconsistent mediation pattern for SPSU (a significant negative indirect association via SA alongside a small, opposite-signed non-significant direct path) should be interpreted cautiously. With single-wave data, such inconsistency may reflect unmeasured third variables (e.g., loneliness, fear of missing out, habitual use, or platform-specific reinforcement), opposing concurrent processes, and/or heterogeneity within social smartphone activities. Future longitudinal research and finer-grained indicators of social smartphone behavior (e.g., active vs. passive social use) would help clarify whether this pattern replicates and under what conditions. The overall direction is broadly consistent with Çelik and Konan (2019) and is compatible with CIUM accounts in which negative affect motivates compensatory use (Elhai et al., 2018; Kardefelt-Winther, 2014; Wolniewicz et al., 2018). Overall, the present findings suggest that perceived support may relate to PSU primarily through anxiety-linked compensatory processes rather than a stable, direct effect (Ding et al., 2022).

Regarding control variables, this study found that higher SSES was associated with lower SA among university students and exhibited a trend toward protective effects on NSPSU, while its direct impact on SPSU was weak and non-significant. This result broadly aligns with the general direction observed in existing research that “lower family socioeconomic status correlates with excessive screen use, while higher social support exerts a protective effect” (Li et al., 2025; Mollborn et al., 2022). Regarding gender, females exhibited significantly higher SA and SPSU levels than males, while gender differences in NSPSU were not pronounced. This aligns with prior findings indicating that “females are more likely to report higher anxiety levels, anxiety symptoms, and problematic social media use” (McLean et al., 2011; Stănculescu and Griffiths, 2022). This suggests that female university students should be a particular focus of interventions targeting SA and SPSU.

## 5 Implications

This study contributes in three main ways. Specifically, it estimates the indirect associations from PSS to both SPSU and NSPSU via SA, helping to clarify how perceived support and social anxiety are jointly patterned with distinct PSU outcomes. By separating social from non-social PSU, it also indicates that SA is a shared risk factor for both types, while the association appears stronger for NSPSU (e.g., gaming, short-video viewing, and information scrolling), consistent with calls to prioritize application- or use-specific patterns rather than treating PSU as a unitary construct (Marino et al., 2021). Finally, the findings provide initial evidence that the “PSS → SA → PSU” pathway reported by Çelik and Konan (2019) may extend to Chinese university students. Taken together, the results offer early cross-cultural support for the co-occurrence of lower perceived support, higher social anxiety, and greater PSU, while falling short of a strict confirmatory replication.

From a practical perspective, prevention and intervention efforts may benefit from a dual focus. On the one hand, institutions can strengthen the “support scaffold” by fostering supportive family and campus environments, increasing approachable teacher–student contact (e.g., advising check-ins or mentoring), and improving access to counseling resources. On the other hand, for students reporting elevated SA alongside higher-risk PSU, support may be more effective when it combines social-anxiety–focused strategies (e.g., cognitive restructuring, graded exposure, guided practice) with emotion-regulation skills training (e.g., mindfulness, distress tolerance, and coping plans for urges to scroll, game, or watch short videos). In addition, evidence from Chinese university samples suggests that physical activity may be a relevant adjunct factor in problematic smartphone use, with findings pointing to mechanisms involving self-control/self-esteem and stress-related processes, and to moderation by exercise type or physical activity (Ke et al., 2024; Wang et al., 2025; Yang et al., 2021; Yang et al., 2019). This approach targets both interpersonal resources and proximal socio-emotional processes, rather than focusing only on screen-time reduction.

In the present sample, informal support was higher than formal support. This pattern suggests that support-building may need to extend beyond informal circles to schools and local communities. Accordingly, institutions could strengthen educators’ supportive roles and broaden low-barrier access to community-based resources for university students.

## 6 Limitations and future directions

Several limitations should be considered. First, this single-university, self-selected online convenience sample may limit generalizability; future studies should replicate the model in multi-site samples drawn from different regions and academic contexts, ideally using probability-based or stratified recruitment. Second, the cross-sectional design precludes causal inference and cannot rule out reciprocal relations among PSS, SA, and PSU; multi-wave longitudinal and intervention designs are needed to establish temporal ordering and to evaluate alternative directional models (e.g., PSU → SA). Third, because all variables were measured via self-report in an online survey, common method variance and related reporting biases may still be present despite the Harman’s test results, and volunteer-selection effects (e.g., differential access and digital literacy) may be present. Where feasible, combining self-reports with behavioral indicators (e.g., screen-time/app-category logs) or intensive longitudinal approaches (e.g., EMA) would strengthen measurement validity and reduce shared-method bias. Fourth, although we distinguished social and non-social PSU, NSPSU likely remains heterogeneous across app domains. Future work should further disaggregate NSPSU (e.g., short-video viewing vs. gaming vs. information browsing) and differentiate active versus passive forms within SPSU to clarify subtype-specific mechanisms. Fifth, while reliability and loadings were generally acceptable, RMSEA indicated only marginal fit for the adapted measurement models; parceling and subscale indicators may have obscured item-level heterogeneity. Accordingly, future studies should test item-level (or alternative latent) measurement models and evaluate measurement invariance across key groups (e.g., gender) before drawing conclusions about group differences or comparing structural paths. Sixth, analyses relied on ML treating 5-point items as quasi-continuous; robust estimators for ordered categorical data (e.g., WLSMV) and sensitivity analyses are warranted to assess the stability of the structural results. Finally, unmeasured third variables and alternative mechanisms (e.g., internalizing symptoms such as loneliness, and self-regulatory factors such as habitual use) as well as lifestyle and family-context factors linked to PSU (e.g., physical activity, self-control, and parental psychological control), may confound or compete with SA; future studies should test competing mediation models and boundary conditions. Despite these constraints, theory-driven cross-sectional tests can still inform mechanism development and hypothesis generation when causal claims are stated conservatively (Spector, 2019).

## 7 Conclusion

In a cross-sectional sample of Chinese university students, perceived social support was associated with lower social anxiety, and social anxiety was associated with higher levels of both social and non-social problematic smartphone use. Indirect associations from perceived social support to both PSU subtypes via social anxiety were statistically significant, with a stronger anxiety–NSPSU link than anxiety–SPSU. These findings support a subtype-sensitive view of PSU and underscore the relevance of social anxiety as a correlate, while longitudinal or experimental research—using finer-grained indicators (e.g., active vs. passive use)—is needed to establish temporal ordering and clarify mechanisms.

## Data Availability

The original contributions presented in the study are included in the article/supplementary material, further inquiries can be directed to the corresponding author.
